# Effects of a Disrupted Blood-Brain Barrier on Cholesterol Homeostasis in the Brain*

**DOI:** 10.1074/jbc.M114.556159

**Published:** 2014-06-27

**Authors:** Ahmed A. Saeed, Guillem Genové, Tian Li, Dieter Lütjohann, Maria Olin, Natalia Mast, Irina A. Pikuleva, Peter Crick, Yuqin Wang, William Griffiths, Christer Betsholtz, Ingemar Björkhem

**Affiliations:** From the ‡ Division of Clinical Chemistry, Department of Laboratory Medicine, Karolinska University Hospital, Karolinska Institute, Huddinge, Stockholm 141 86, Sweden,; the § Department of Biochemistry, Faculty of Medicine, University of Khartoum, 11111 Khartoum, Sudan,; the ¶ Department of Medical Biochemistry and Biophysics, Karolinska Institute, 171 77 Stockholm, Sweden,; the ‖ Institute of Clinical Chemistry and Clinical Pharmacology, University of Bonn, D-53127 Bonn, Germany,; the ** Department of Ophthalmology and Visual Sciences, Case Western Reserve University, Cleveland, Ohio 44106, and; the ‡‡ Institute of Mass Spectrometry, College of Medicine, Swansea University, Swansea SA2 8PP, United Kingdom

**Keywords:** Brain Metabolism, Cholesterol Regulation, Hydroxylase, Neurodegeneration, Sterol

## Abstract

The presence of the blood-brain barrier (BBB) is critical for cholesterol metabolism in the brain, preventing uptake of lipoprotein-bound cholesterol from the circulation. The metabolic consequences of a leaking BBB for cholesterol metabolism have not been studied previously. Here we used a pericyte-deficient mouse model, *Pdgfb^ret/ret^*, shown to have increased permeability of the BBB to a range of low-molecular mass and high-molecular mass tracers. There was a significant accumulation of plant sterols in the brains of the *Pdgfb^ret/ret^* mice. By dietary treatment with 0.3% deuterium-labeled cholesterol, we could demonstrate a significant flux of cholesterol from the circulation into the brains of the mutant mice roughly corresponding to about half of the measured turnover of cholesterol in the brain. We expected the cholesterol flux into the brain to cause a down-regulation of cholesterol synthesis. Instead, cholesterol synthesis was increased by about 60%. The levels of 24(*S*)-hydroxycholesterol (24*S*-OHC) were significantly reduced in the brains of the pericyte-deficient mice but increased in the circulation. After treatment with 1% cholesterol in diet, the difference in cholesterol synthesis between mutants and controls disappeared. The findings are consistent with increased leakage of 24*S*-OHC from the brain into the circulation in the pericyte-deficient mice. This oxysterol is an efficient suppressor of cholesterol synthesis, and the results are consistent with a regulatory role of 24*S*-OHC in the brain. To our knowledge, this is the first demonstration that a defective BBB may lead to increased flux of a lipophilic compound out from the brain. The relevance of the findings for the human situation is discussed.

## Teaser

**Background:** The role of the blood-brain barrier for cholesterol homeostasis in the brain is not known.

**Results:** Significant influx of cholesterol into the brain and increased efflux of 24(*S*)-hydroxycholesterol were observed in mice with a defect blood-brain barrier.

**Conclusion:** A defect blood-brain barrier increases permeability for steroid flux in both directions.

**Significance:** Elucidation of the role of the blood-brain barrier for brain cholesterol turnover.

## Introduction

The blood-brain barrier (BBB)^2^ is a combination of physical barriers, enzymes, and transporters. At the level of endothelial cells, neuronal cells, glial cells, and pericytes are of regulatory importance. Pericytes seem to be of particular importance for the permeability of the BBB (1, 2). It was recently shown that pericyte deficiency increases the permeability of BBB to water and to a range of low- and high-molecular mass tracers (2). The increased permeability was found to be dependent upon endothelial transcytosis. It was also recently reported that lack of the cholesterol transporter ApoE or the presence of the isoform ApoE4 leads to BBB dysfunction (3). The latter dysfunction is mediated by activation of a cyclophilin A pathway in pericytes.

The BBB is of particular importance for cholesterol metabolism in the brain (for reviews, see Refs. 4 and 5). This barrier effectively prevents uptake of lipoprotein-bound cholesterol from the circulation, and *de novo* synthesis is considered to be responsible for practically all cholesterol in this organ. The independence of the isolated pool of cholesterol in the brain is likely to reflect a high need for constancy in the cholesterol content of membrane and myelin, a constancy that would be difficult to keep if brain cholesterol had been exchangeable with lipoprotein cholesterol in the circulation. The brain does not seem to respond to many of the regulatory mechanisms operating in maintaining extracerebral cholesterol homeostasis. The half-life of the bulk of cholesterol in the brain has been estimated to be about 5 years and 4 months in humans and mice, respectively (4, 5). In contrast, the half-life of cholesterol in human circulation is only a few days.

In contrast to cholesterol itself, some side-chain hydroxylated metabolites of cholesterol are able to pass the blood-brain barrier (6). It has been shown that about two-thirds of the synthesis of cholesterol in the brain is balanced by neuronal formation of 24(*S*)-hydroxycholesterol (24*S*-OHC) that is fluxing across the BBB into the circulation (7, 8). Another side-chain hydroxylated oxysterol, 27-hydroxycholesterol (27-OHC), is taken up by the brain and converted into a steroid acid, 7α-hydroxy-3-oxo-4-cholestenoic acid, that is fluxing out from the brain into the circulation (9). In view of the potent effect of 27-OHC on a number of systems that are of importance in connection with neurodegeneration (for a review, see Ref. 10), the latter pathway may be regarded as a detoxification. Both 24*S*-OHC and 27-OHC are efficient inhibitors of cholesterol synthesis *in vitro* (11, 12). Recent studies with transgenic mouse models are consistent with the possibility that both the flux of 27-OHC into the brain and the size of the pool of 24*S*-OHC in the brain are of regulatory importance for cholesterol synthesis (13).

Very little is known about the consequences of a disrupted BBB on cholesterol homeostasis in the brain. Increased permeability of the BBB may lead to a significant flux of cholesterol into the brain and/or loss of cholesterol from the brain into the circulation. With respect to the side-chain oxidized oxysterols, a disrupted BBB would be expected to increase the flux of 27-OHC into the brain and possibly also the flux of 24*S*-OHC out from the brain.

In the present work, we have studied the consequences of pericyte deficiency for brain cholesterol homeostasis in a mouse model, *Pdgfb^ret/ret^*. This mouse model has been shown to have increased permeability of the BBB to water and a range of both low- and high-molecular mass tracers. These mice have severe glomerular and retinal defects but have a normal life span (14). The leaking BBB in these mice would be expected to lead to an influx of cholesterol from the circulation with consequences for cholesterol homeostasis in the brain. It is shown here that *Pdgfb^ret/ret^* mice have a slightly increased synthesis of cholesterol in the brain and that at least part of this increase is likely to be due to increased loss of intracerebral 24*S*-OHC.

## EXPERIMENTAL PROCEDURES

### 

#### 

##### Animals

The studies were performed with transgenic mice (*Pdgfb^ret/ret^*) lacking the PDGF-B retention motif and their littermate controls (*Pdgfb^ret^*^/+^), backcrossed at least seven generations against C57Bl/6 and identified by PCR genotyping. The heterozygote controls are shown to have normal BBB function (2). Only male mice were used with an age of about 10 weeks (8–12 weeks). Three types of diets were used in the study: chow diet, 1% cholesterol diet, and chow diet labeled with 0.3% cholesterol-*d*_6_. All of the mice were sacrificed between 8 and 10 a.m.

##### Ethical Considerations

All experimental procedures in this study were in compliance with the National Institutes of Health Guide for Care and Use of Laboratory Animals and the European Communities Council Directive of 24 November 1986 (86/609/EEC) and approved by the Northern Stockholm Research Animal Ethics Committee.

##### Lipid Extraction and Analysis

Lipids were extracted according to the Folch method. Different volumes of Folch solution (chloroform/methanol, 2:1 (v/v)) were added to half of a brain and other different organs according to their weights. After 24 h at room temperature, extracts were transferred to new vials and evaporated under argon. After evaporation, the extracts were redissolved in appropriate volumes of Folch and stored at −20 °C until required.

Sterols and oxysterols were measured by gas chromatography-mass spectrometry (GC-MS) using deuterium-labeled internal standards as described previously (15, 16). 24(*R*)- and 24(*S*)-hydroxycholesterols were analyzed as their Girard P hydrazone derivatives by liquid chromatography-mass spectrometry (LC-MS*^n^*) as described (17). The two isomers separate chromatographically on the reversed phase LC column.

##### RNA Preparation and Real-time PCR

Total RNA was prepared using TRIzol reagent (Invitrogen) according to the manufacturer's protocol. RNA (1 μg) was transcribed into cDNA using a high-capacity cDNA reverse transcription kit (Applied Biosystems, Carlsbad, CA). The cDNA obtained was diluted 1:10 in RNase-free H_2_O. Real-time PCR was performed with 5 μl of cDNA and 12.5 μl of SYBR Green Mastermix (Applied Biosystems). The forward and reverse primers are shown in Table 1. All values were normalized to hypoxanthine phosphoribosyltransferase mRNA concentrations for hepatic analyses.

**TABLE 1:** **Forward and reverse primers used to measure the relative expression of different genes**

Gene	Sequence (5′–3′)	Concentration
		*nm*
*Hprt* forward	GGT GAA AAG GAC CTC TCG AAG TG	200
*Hprt* reverse	ATA GTC AAG GGC ATA TCC AAC AAC A	200
*Hmgcr* forward	CCG GCA ACA ACA AGA TCT GTG	200
*Hmgcr* reverse	ATG TAC AGG ATG GCG ATG CA	200
*Hmgcs1* forward	CTC TGT CTA TGG TTC CCT GGC T	200
*Hmgcs1* reverse	TCC AAT CCT CTT CCC TGC C	200
*Abca1* forward	CCC AGA GCA AAA AGC GAC TC	200
*Abca1* reverse	GGT CAT CAT CAC TTT GGT CCT TG	200
*Abcg1* forward	GCTGAAGAGGACTCCGCCT	200
*Abcg1* reverse	GAGGATGCAGAACTGGGTGAG	200
*Srebp1c* forward	GGAGCCATGGATTGCACATT	200
*Srebp1c* reverse	GGCCCGGGAAGTCACTGT	200
*Srebp2* forward	GCG TTC TGG AGA CCA TGG A	700
*Srebp2* reverse	ACA AAG TTG CTC TGA AAA CAA ATC	700
*ApoE* forward	GCA GGC GGA GAT CTT CCA	200
*ApoE* reverse	CCA CTG GCG ATG CAT GTC	200
*Cyp7b1* forward	CCT CTT TCC TCC ACT CAT ACA CAA	200
*Cyp7b1* reverse	GAA CCG ATC GAA CCT AAA TTC CTT	200
*Cyp27* forward	GCC TTG CAC AAG GAA GTG ACT	200
*Cyp27* reverse	CGC AGG GTC TCC TTA ATC ACA	200
*Fas* forward	GGCATCATTGGGCACTCCTT	200
*Fas* reverse	GCTGCAAGCACAGCCTCTCT	200
*Cyp46* forward	AACCATCTGGCATTCACAGTGA	200
*Cyp46* reverse	GGAACCGACAACCTCATCCA	200

##### Western Blots

Microsomes prepared from brains of the *Pdgfb^ret/ret^* mice and their controls were subjected to electrophoresis (20–25 μg/lane) on a 10% SDS or BisTris polyacrylamide gel and transferred to nitrocellulose membranes. The membranes were incubated for 2 h at room temperature in blocking buffer (5% milk in phosphate-buffered saline, 0.05% Tween) followed by incubation overnight at +4 °C with an anti-CYP46 antibody (a generous gift from Prof. D. Russell, University of Texas Southwestern Medical Center, Dallas, TX) or an antibody directed toward Abca1 (anti-Abca1 antibody ab 18180 from Abcam). Goat anti-rabbit IgG or goat anti-mouse IgG coupled with horseradish peroxidase (Pierce) was used as a secondary antibody with incubation at room temperature for 2 h. The membranes were incubated in Super Signal West Dura extended duration substrate (product number 34075 (Pierce) according to the manufacturer's instructions. The signal (around 50 kDa for CYP46 and 250 kDa for ABCA1) was determined using Universal hood II equipment (Bio-Rad).

##### Experiments with Deuterated Water and Calculation of Fractional Synthetic Rate

The mice were exposed to drinking water containing 10% deuterium water for a period of 11 days before sacrifice (18). In addition to measurements of deuterium content in brain cholesterol, the deuterium content was measured in body fluid (serum) of the mice as described (18). From the deuterium enrichment in cholesterol and body water, the fractional synthetic rate could be calculated (18).

##### Statistics

Gene expression data are expressed as mean ± range as described by Livak *et al.* (19). Sterol determinations are expressed as means ± S.E. Statistical comparisons were performed using the unpaired Student's *t* test.

## RESULTS

### 

#### 

##### Experiments with Deuterium-labeled Cholesterol

**FIGURE 1.**
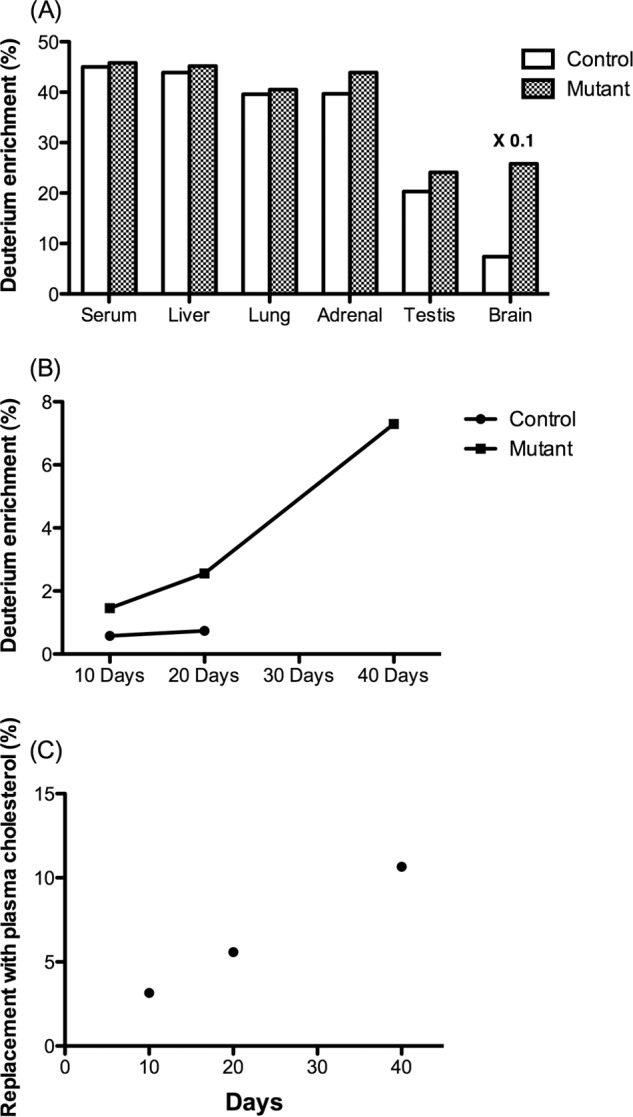
*A*, enrichment of cholesterol-*d*_6_ in cholesterol isolated from serum and different organs in a control mouse and a mutant mouse treated with 0.3% cholesterol-*d*_6_ in the diet for 20 days. *B*, enrichment of cholesterol-*d*_6_ in cholesterol isolated from the brains of three mutants and three control mice treated with 0.3% cholesterol-*d*_6_ for 10, 20, or 40 days, respectively. *C*, replacement of brain cholesterol with serum cholesterol was calculated at the different time points with use of the percentage incorporation of deuterium in plasma cholesterol. Data are from GC-MS analysis.

Fig. 1*A* shows the enrichment of deuterium in serum cholesterol and in cholesterol isolated from different organs of a mutant (*Pdgfb^ret/ret^*) mouse and a control (*Pdgfb^ret^*^/+^) mouse treated with 0.3% cholesterol-*d*_6_ in the diet for 20 days. The enrichment of deuterium was found to be about 45% in serum with very little difference between the mutant and the control. The situation was about the same in cholesterol isolated from liver, lung, and adrenals. A barrier function would be expected to give lower enrichment of deuterium in the cholesterol pool, and in accordance with this, lower enrichment was found in testis cholesterol, and markedly lower enrichment was found in brain cholesterol. If the barrier function is defective, higher enrichment of deuterium would be expected in the cholesterol isolated from the mutant than from the control. In accordance with this, slightly higher enrichment of deuterium was found in the testis from the mutant (probably due to a defective testis-blood barrier). Markedly higher enrichment was found in cholesterol isolated from the brains of the mutant as compared with the control, suggesting a serious defect in the blood-brain barrier. The data shown in Fig. 1*A* are from one single experiment and are representative of two independent experiments.

Because it is almost impossible to remove all of the blood from the brain, it is likely that a substantial part of the small enrichment of deuterium in cholesterol isolated from the brains of the above mice is due to contamination with blood containing cholesterol-*d*_6_. A flux of cholesterol-*d*_6_ into the brain would be expected to lead to a time-dependent increase in the accumulation of isotope in the brain, whereas this is not the case with the cholesterol-*d*_6_ present due to contamination with blood. In order to estimate the rate of the flux from the circulation into the brain, we compared the accumulation of cholesterol-*d*_6_ in the brain after 10 and 20 days of dietary treatment with cholesterol-*d*_6_. The enrichment of deuterium in brain cholesterol in the control increased from 0.6 to 0.7% between days 10 and 20. The corresponding figures for the mutant were 1.5 and 2.6%, respectively (Fig. 1*B*). In an additional experiment, we treated a mutant mouse with 0.3% cholesterol-*d*_6_ for 40 days. The enrichment of deuterium in brain cholesterol was 7.3%. In the first two experiments, the enrichment of deuterium in circulating cholesterol was about 50%, and in the latter experiment, it was about 70%. Fig. 1*C* shows the enrichment of deuterium in the brain cholesterol that could be expected if the enrichment of deuterium in circulating cholesterol had been 100%. The higher content of deuterium in circulating cholesterol in the long term experiments (40 days) than in the short term experiments (10 and 20 days) may be a consequence of a slow exchange of endogenous cholesterol in different extracerebal pools with exogenous cholesterol-*d*_6_.

The data shown in Fig. 1, *B* and *C*, are from three separate independent experiments with one mutant and one control mouse in each. The very high costs of the isotope prevented us from performing additional experiments allowing statistical evaluations. However, the linear enrichment of isotope with time and the marked difference between the controls and the mutants allow safe conclusions also in the absence of statistical evaluation.

**FIGURE 2.**
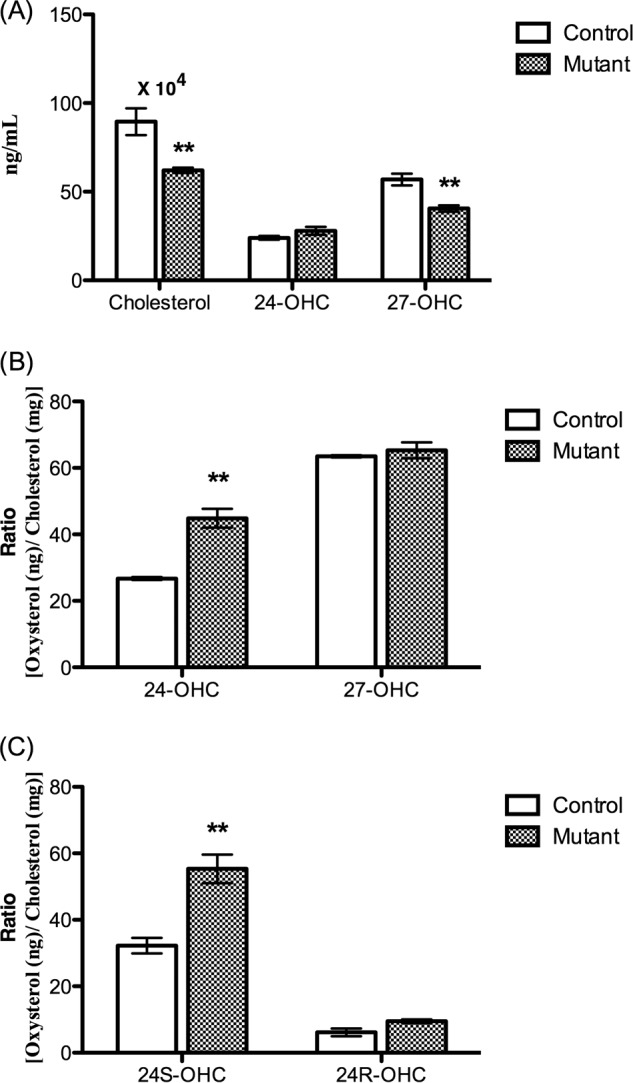
*A* and *B*, levels of cholesterol, 24(*S*)-hydroxycholesterol, and 27-hydroxycholesterol in serum from *Pdgfb^ret/ret^* mice and their controls (absolute levels) (*A*) and cholesterol-correlated levels of the oxysterols (*B*) (data from GC-MS analysis). *C*, cholesterol-correlated levels of 24(*R*)- and 24(*S*)-hydroxycholesterol in serum from three control mice and three *Pdgfb^ret/ret^* mice (age 10 weeks, *n* = 3). Data are from LC-MS*^n^* analysis. *Error bars*, S.E.

It is concluded that most or all cholesterol-*d*_6_ present in brain cholesterol in the control is likely to be due to a contamination with blood cholesterol. Thus, there is no significant flux of cholesterol-*d*_6_ into the brain in the controls. In the mutant, however, there is a significant flux from the circulation into the brain between day 10 and 20 of the treatment. This enrichment was 1.1% under the conditions employed. Because the enrichment of serum cholesterol was 45% rather than 100%, the data are consistent with a flux of cholesterol from the circulation into the brain corresponding to replacement of about 2.2% of the total cholesterol pool with plasma cholesterol during the 10 days of treatment. The difference in replacement of brain cholesterol between day 10 and day 40 was calculated to be about 8.6%. The time course of the accumulation of cholesterol-*d*_6_ suggests that there is an increasing leakage of cholesterol into the brain with time. Fig. 2*B* shows the percentage of the replacement of brain cholesterol by plasma cholesterol in the mutant mice at different time points. By day 40, it is estimated that more than 10% of total brain cholesterol in these mice originates from the plasma.

The incorporation of cholesterol-*d*_6_ was also measured in cholesterol in different parts of the brain in the long term experiment (40 days of feeding with 0.3% cholesterol-*d*_6_). The incorporation in cholesterol was as follows: hippocampus, 9.4%; cortex, 8.3%; cerebellum, 3.7%; corpus callosum, 6.0%; pituitary, 47%. It should be noted that, in contrast to the other parts of the brain, the pituitary is not protected by the blood-brain barrier.

##### Cholesterol and Oxysterol Levels in Circulation of the Pdgfb^ret/ret^ Mice

Fig. 2*A* summarizes the levels of cholesterol and the oxysterols 24-OHC and 27-OHC in the circulation of the *Pdgf^ret/ret^* mice and their controls. The cholesterol levels as well as the levels of 27-hydroxycholesterol were significantly lower in the mutated mice. The levels of 24-OHC were slightly higher. In view of the fact that the oxysterols are transported by cholesterol-containing lipoproteins in the circulation (20), the ratio between the oxysterols and cholesterol should be used in a comparison. As shown in Fig. 2*B*, the cholesterol-correlated levels of 24-OHC in serum were significantly higher in the mutated mice than in the controls. The cholesterol-correlated levels of 27-OHC were similar in the mutants and the controls.

**FIGURE 3.**
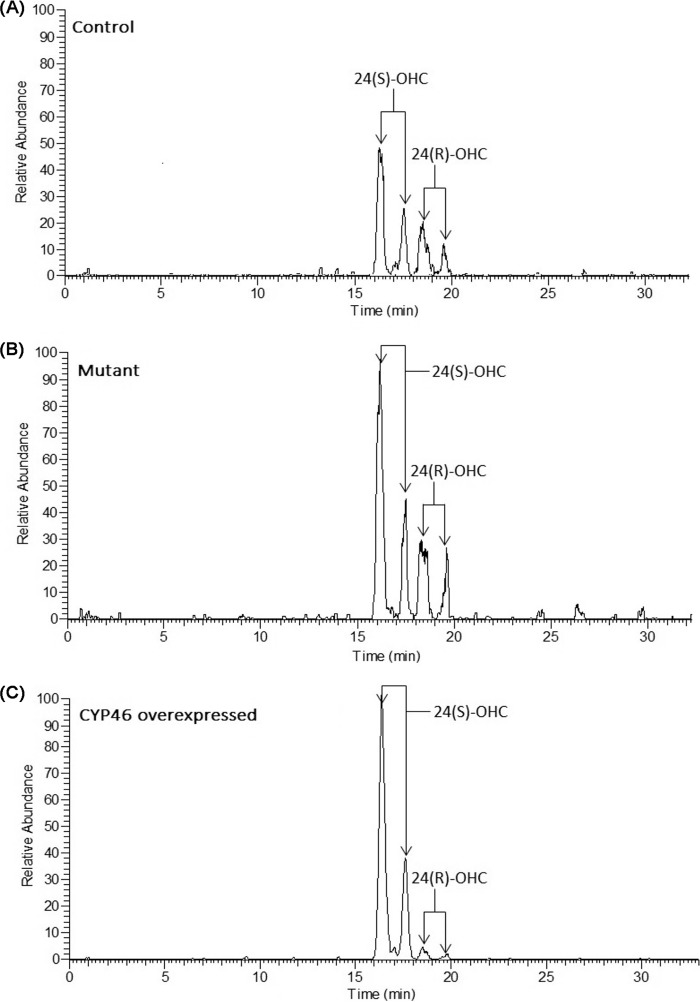
**Typical reconstructed ion chromatograms (*RIC*) obtained in the analysis of 24(*R*)- and 24(*S*)-hydroxycholesterols in serum from a control mouse (*A*), a *Pdgfb^ret/ret^* mouse (*B*), and a CYP46 transgenic mouse (40) (*C*) using LC-MS*^n^* after derivatization to Girard P hydrazones (see “Experimental Procedures”).** The reconstructed ion chromatograms were generated from multiple reaction-monitoring transitions specific for 24-OHC isomers (*m/z* 534 → 455 → 353).

**FIGURE 4.**
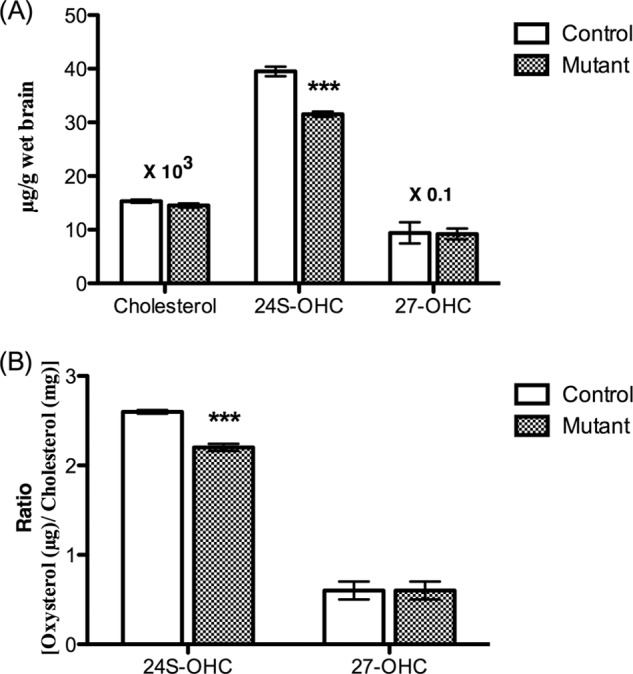
**Levels of cholesterol and oxysterols in the brains of *Pdgfb^ret/ret^* mice and their controls (age 10 weeks, *n* = 6) related to brain weight (*A*) and oxysterols related to cholesterol (*B*).** Data are from GC-MS analysis. *Error bars*, S.E.

In humans, all of the 24-hydroxycholesterol present in the circulation originates from the brain, and it is only the 24(*S*)-stereoisomer that is formed (21). In mice, almost half of the 24-hydroxycholesterol present in the circulation originates from an organ(s) or tissues other than the brain (22). We have found that 24(*S*)- is the only stereoisomer of 24-OHC present in the mouse brain, whereas the circulation contains a mixture of the 24(*S*)- and the 24(*R*)-isomer.^3^ Most of the 24(*S*)-isomer in the circulation must originate from the brain. It is difficult to separate the 24(*S*)- and 24(*R*)-isomers by gas chromatography-mass spectrometry of the trimethylsilyl ether derivatives. An LC-MS*^n^* method has recently been developed capable of separating the two isomers after oxidation with cholesterol oxidase and conversion into Girard hydrazones (17). We analyzed serum from three control mice and three *Pdgf^ret/ret^* mice with this method. Fig. 3 shows typical chromatograms obtained. As shown, both 24(*R*)- and 24(*S*)-epimers give two peaks corresponding to the *syn-* and *anti*-conformers of the derivative (Fig. 4). For reasons of comparison, we show the chromatogram obtained in the analysis of serum from a mouse with overexpressed CYP46. In this mouse, the situation is similar to the human situation, with 24*S*-OHC greatly dominating. The levels of 24*S*-OHC increased from 32 ± 2 ng/mg cholesterol in the control mice to 55 ± 4 ng/mg cholesterol in mutant mice (*p* < 0.01). The levels of 24(*R*)-hydroxycholesterol increased from 6.1 ± 1.1 ng/mg cholesterol in the control mouse to 9.4 ± 0.5 ng/mg cholesterol in the mutant mouse (*p* > 0.05). Fig. 2*C* shows the cholesterol-correlated levels of 24(*S*)- and 24(*R*)-hydroxycholesterol in the circulation of the control mice and the *Pdgf^ret/ret^* mice.

The above data are consistent with a higher flux of 24(*S*)-OHC from the brain into the circulation in the mutated mice. Part of the increase in 24(*S*)- and 24(*R*)-hydroxycholesterol may, however, be due to increased production of these oxysteroids in extracerebral tissues. Oxysterols formed extracerebrally in mice treated with cholesterol-*d*_6_ can be expected to contain deuterium to about the same extent as circulating cholesterol. In contrast, the cerebral formation of 24*S*-OHC would be expected to contain very little deuterium (7% in the experiment in which a mutant mouse was treated with cholesterol-*d*_6_ for 40 days). The content of deuterium in 24(*S*)-hydroxycholesterol in the circulation of the above mouse was found to be 18%. After correction for the content of deuterium in the brain cholesterol (7%) and the fact that the enrichment of deuterium in circulating cholesterol was about 70% rather than 100%, it can be concluded that 15–20% of the 24(*S*)-hydroxycholesterol in the circulation originates from extracerebral sources. The content of deuterium in 24R-hydroxycholesterol was found to be 90%. After correction for the enrichment of deuterium in the cholesterol in the circulation (about 70%), it can be concluded that all of the 24(*R*)-hydroxycholesterol in the circulation originates from extracerebral sources.

For reasons of comparison, we also measured the deuterium content in 27-OHC in the circulation of the control mouse and the mutant mouse treated with cholesterol-*d*_6_ for 40 days. The deuterium content was 37 and 33%, respectively. Because one of the six deuterium atoms in cholesterol-*d*_6_ is lost in connection with its conversion into 27-OHC, the product will contain five rather than six atoms of deuterium. It has been shown that there is a significant isotope effect in connection with replacement of one deuterium in the steroid side chain of cholesterol-*d*_6_ (23). Thus, the deuterium content in *d*_5_-labeled 27-OHC is lower than had been the case in the absence of an isotope effect.

##### Cholesterol and Oxysterol Levels in the Brains of Pdgfb^ret/ret^ Mice

Fig. 4 summarizes the levels of cholesterol and the oxysterols in the brains of the *Pdgf^ret/ret^* mice. The cholesterol levels (related to brain weight) were found to be similar to those in the brains of the mutant mice (Fig. 4*A*). The situation was similar when the levels were related to protein (results not shown). The level of 24(*S*)-OHC was significantly lower in the brains of the mutant mice than in the controls, and this was the situation also when correlated to protein or cholesterol (Fig. 4*B* shows the cholesterol-related levels). The levels of 27-OHC were not different in the brains of the mutants and the controls, regardless of whether related to brain weight, protein, or cholesterol.

##### Levels of Plant Sterols in the Brains of the Pdgfb^ret/ret^ Mice

**FIGURE 5.**
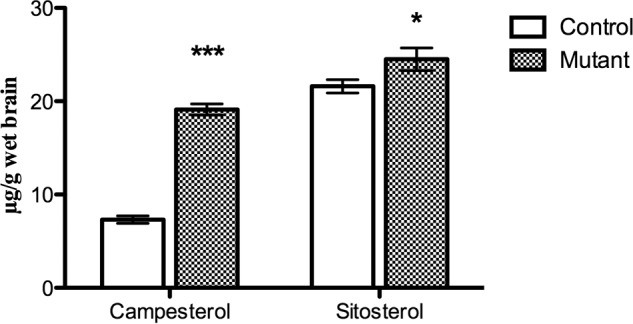
**Levels of plant sterols in the brain of the *Pdgfb^ret/ret^* mice and the controls (age 10 weeks, *n* = 6).** Data are from GC-MS analysis. *Error bars*, S.E.

Plant sterols may accumulate to some extent in the brain, probably as a consequence of the fact that in contrast to cholesterol, these steroids cannot be eliminated by 24(*S*)-hydroxylation (24). A defective blood-brain barrier would be expected to lead to an increase in such accumulation. As shown in Fig. 5, there was a significant accumulation of both campesterol and sitosterol in the brains of the mutated mice. The degree of accumulation of sitosterol was lower than that of campesterol.

##### Levels of Cholesterol Precursors in the Brains of Pdgfb^ret/ret^ Mice

**FIGURE 6.**
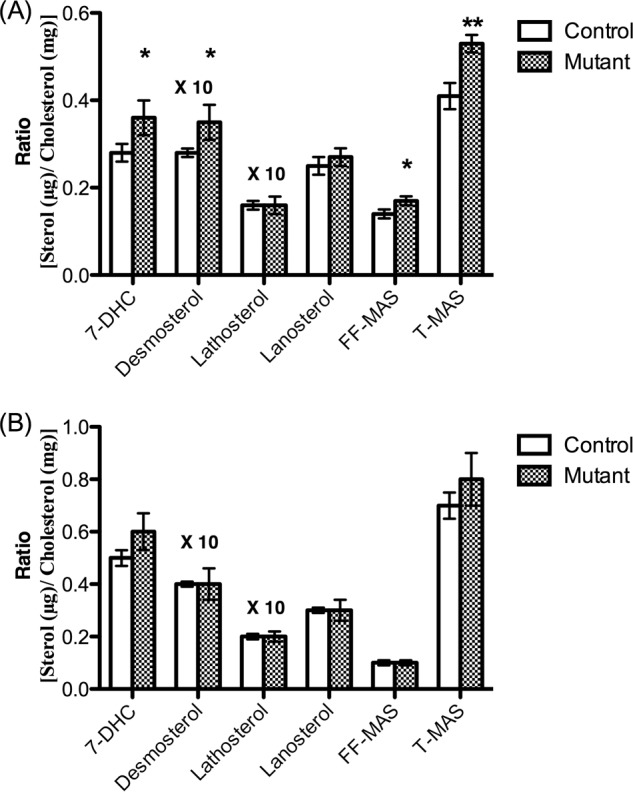
**Cholesterol-correlated levels of cholesterol precursors in the brains of the *Pdgfb^ret/ret^* mice and their controls (*n* = 6) on regular chow diet (*A*) and on 1% cholesterol diet (*n* = 4) (*B*).** Data are from GC-MS analysis. *FF-MAS*, follicular fluid meiosis-activating sterol, 4,4-dimethyl-5α-cholesta-8,14,24-trien-3β-ol; *T-MAS*, testicular meiosis-activating sterol, 4,4-dimethyl-5α-cholesta-8,24-dien-3β-ol; *7-DHC*, 7-dehydrocholesterol. *Error bars*, S.E.

The ratio between cholesterol precursors and cholesterol is generally used as a marker for cholesterol synthesis. As shown in Fig. 6*A*, the cholesterol-related levels of the cholesterol precursors desmosterol, testicular meiosis-activating sterol (*T-MAS*), follicular fluid meiosis-activating sterol (*FF-MAS*), and 7-dehydrocholesterol (*7-DHS*) were significantly higher in the brains of the mutated mice than in the controls, suggesting an increased rate of cholesterol synthesis.

##### Expression of Key Genes in Cholesterol Homeostasis

**FIGURE 7.**
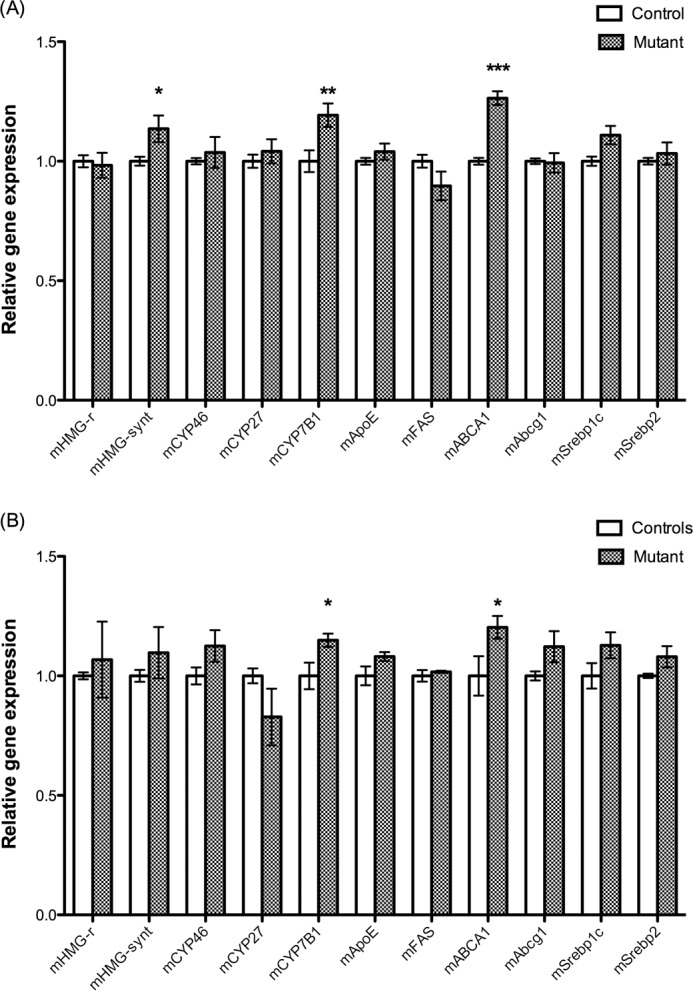
*A*, expression of key genes in cholesterols homeostasis in the untreated *Pdgfb^ret/ret^* mice and their controls (*n* = 6). *B*, expression of the same genes in the *Pdgfb^ret/ret^* mice (*n* = 4) treated with 1% cholesterol and their controls (*n* = 4) (*B*). *Error bars*, S.E.

The increased levels of cholesterol precursors in the brain suggest an increased rate of synthesis of cholesterol. Such increased synthesis could be expected to be combined with increased levels of HMG-CoA reductase (*Hmgcr*) and/or HMG-CoA synthase (*Hmgcs1*). The mRNA levels of HMG-CoA synthase were slightly but significantly higher in the mutants as compared with the controls (Fig. 7*A*). The mRNA level of *Srebp2* was not significantly increased, and this was the case also with *Cyp46*. Expression of two LXR-regulated genes, *Cyp7b1* and *Abca1*, were significantly increased in the mutant. Other LXR-regulated genes (*Fas*, *Abcg1*, and *Apoe*) were not affected by the mutation.

##### Western Blot of the Cholesterol 24(S)-Hydroxylase (Cyp46) and Abca1

The gene encoding the cholesterol 24(*S*)-hydroxylase was not up-regulated in the mutant mice, but in view of the importance of this enzyme for cholesterol homeostasis, we also measured the protein content of this hydroxylase. The protein content in brain microsomes from the mutant mice and the controls was not significantly different. The ratio between cyp46 and β-actin protein was thus 0.040 ± 0.005 in the analysis of microsomes from the controls and 0.038 ± 0.009 in the analysis of microsomes from the mutants (*n* = 6 ± 6, *p* > 0.05). The LXR target gene *Abca1* was up-regulated by about 20% (Fig. 7); thus, we measured the corresponding protein levels by Western blots. The ratio between Abca1 and β-actin protein was 0.18 ± 0.02 in the analysis of microsomes from the controls and 0.33 ± 0.02 in the corresponding analysis of the mutants (*n* = 6 ± 6, *p* < 0.002).

##### Evidence for Increased Cholesterol Synthesis in the Brains of the Pdgfb^ret/ret^ Mice

The increased levels of cholesterol precursor in the brains of the mutant mice and the higher mRNA levels of HMG-CoA synthase suggest a higher rate of synthesis in these mice. In order to confirm this, we utilized a technique based on incorporation of deuterium into brain cholesterol from deuterated water (18). Mice were exposed to 10% D_2_O in deuterated water for 11 days before sacrifice. The incorporation of deuterium was found to be 6.3 ± 0.3% in the brain cholesterol from the control mice (*n* = 6) and 9.0 ± 0.4% in the brain cholesterol from the mutants (*p* < 0.001, Student's *t* test). From this incorporation of deuterium and the enrichment of deuterium measured in the body water, the fractional synthetic rate was calculated to be 0.29 ± 0.03%/24 h in the controls and 0.47 ± 0.05%/24 h in the mutants (*p* < 0.05, Student's *t* test).

##### Effects of Treating the Pdgfb^ret/ret^ Mice and Their Controls with 1% Cholesterol in Diet

If the blood-brain barrier is leaking, one would expect a higher influx of cholesterol into the brain after treatment of the mice with cholesterol. Such an increased influx would be expected to reduce cholesterol synthesis in the brain. As shown in Fig. 6*B*, a dietary load of 1% cholesterol during 10 days normalized the levels of the cholesterol precursors. Also, the mRNA levels of HMG-CoA synthase were normalized as a consequence of the cholesterol load (Fig. 7*B*). The level of 24-OHC in the brain was still significantly lower in the brains of the mutated mice as compared with the controls (results not shown). For reasons of comparison, we also measured the mRNA levels of HMG-CoA synthase in the livers of the mutant and the control mice treated with cholesterol. The cholesterol treatment caused a reduction by about 88% in the livers of both the mutant and the control mice (results not shown).

## DISCUSSION

The *Pdgfb^ret/ret^* mouse model was used here to study the consequences of a leaking blood-brain barrier for cholesterol homeostasis in the brain. It should be emphasized that the model is complicated and that also extracerebral cholesterol homeostasis is affected. Pericyte deficiency leads to increased vascular permeability not only in the brain but also in the liver (25). In preliminary studies, we have shown that the *Pdgfb^ret/ret^* mice have an abnormal lipoprotein pattern in the circulation with low levels of HDL. Most probably this is due to increased leakage of this lipoprotein from the microvessels. There is a tendency toward increased cholesterol content in extracerebral organs, possibly because of increased leakage of lipoprotein-bound cholesterol from the microvessels.^3^

We had expected the leaking blood-brain barrier to result in an increased flux of lipoprotein-bound cholesterol from the circulation into the brain. In accordance with this, we found that enrichment of the cholesterol in the circulation with deuterium led to a modest accumulation of deuterium-labeled cholesterol in the brain. The enrichment of deuterium in brain cholesterol during the 40 days of treatment corresponds to a daily replacement of about 0.25% of the pool in the mutants. Given our observation that the fractional synthetic rate of brain cholesterol was found to be 0.47%/24 h in the mutant mouse, it is evident that the flux of cholesterol into the brain is about half of the synthesis.

The slight increase in enrichment of cholesterol-*d*_6_ in the brain of the control, from 0.6 to 0.7% during day 10 and day 20 of the treatment, is not significant. If there is a flux of cholesterol from the circulation into the brain under normal conditions, this flux must be very low. This finding is in accord with previous *in vivo* studies with different animal models (21, 26–28) and in one study with humans (29).

Evidence has been presented that endothelial cells contain a low density lipoprotein (LDL) receptor (30). It has also been shown that this receptor is able to mediate transcytosis of LDL across an *in vitro* model of the blood-brain barrier with cultured endothelial cells on one side of a filter and astrocytes on the opposite side (31). Some results also suggest a role of caveolae in the transcellular transport of LDL across the brain endothelium (31). Thus, it is evident that there is a potential for receptor-mediated transcytosis across brain endothelial cells. The present and previous experiments suggest, however, that such a transcytosis is not of importance under normal *in vivo* conditions. Pericytes have been shown to inhibit transcytosis of different low- and high-molecular traces across the blood-brain barrier (2), and the results of the present study suggest that this is likely to be the case also for transcytosis of LDL and LDL-cholesterol across the BBB.

The increased flux of cholesterol from the circulation into the brain of the mutant mice would be expected to lead to a down-regulation of cholesterol synthesis. Surprisingly, the opposite was found. Cholesterol synthesis in the brains of untreated *Pdgfb^ret/ret^* mice was thus found to be increased as judged from three different observations: 1) increased levels of cholesterol precursors, 2) slightly increased mRNA levels of HMG-CoA synthase, and 3) increased fractional synthetic rate of brain cholesterol, as demonstrated with the D_2_O technique. It is evident that the increased flux of cholesterol itself into the brain of the mutant is not able to down-regulate cholesterol synthesis. Some other factor(s) related to the leaking BBB may cause a stimulation of brain cholesterol synthesis.

A flux of lipoprotein-bound cholesterol from the circulation into the brain must be dependent upon its plasma concentration. The fact that the levels of cholesterol were reduced in the circulation of the untreated mutant mice may be part of the explanation for the relatively modest flux of cholesterol into the brains of these mice. The finding that treatment of the mutants with dietary cholesterol normalized cholesterol synthesis in relation to the controls is consistent with an increased flux of cholesterol into the brains of the *Pdgfb^ret/ret^* mice when the levels of cholesterol increase in the circulation.

The increased cholesterol synthesis in the brain of the untreated *Pdgfb^ret/ret^* mouse is likely to be a compensation for a loss of either cholesterol itself or a regulating metabolite. The concentration of cholesterol itself in the brains of the untreated *Pdgfb^ret/ret^* mice was not significantly changed. The level of 24-OHC, the dominating oxysterol in the brain, was, however, significantly reduced in relation to tissue weight, cholesterol, and protein. The reduction was not due to reduced activity of the cholesterol 24(*S*)-hydroxylase because the mRNA levels and protein levels corresponding to this enzyme were unaffected by the mutation. 24-OHC is an efficient suppressor of cholesterol synthesis *in vitro* (11, 12), and we recently showed that reduced levels of 24-OHC in the brain as a consequence of increased metabolism result in increased cholesterol synthesis (13). Increased flux of 24-OHC from the brain into the circulation would be expected to lead to increased levels in the circulation. Utilizing data from the experiment with cholesterol-*d*_6_, we could demonstrate that a least a substantial part of the increased level of 24-OHC in the circulation of the mutant mice is due to an increased flux of this oxysterol from the brain.

**FIGURE 8.**
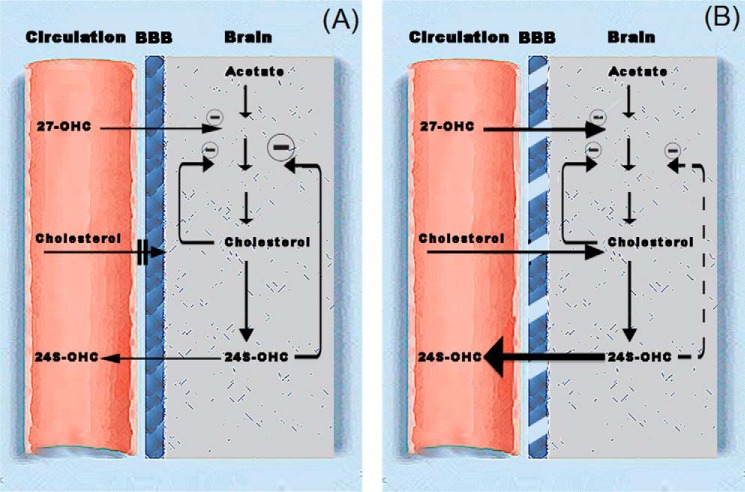
**Theoretical model for the mechanism of regulation of cholesterol homeostasis in the brain.**

In a previous work from our laboratory, we presented a theoretical model for cholesterol homeostasis in the brain that is shown in Fig. 8. This model is consistent with previous *in vitro* results (11, 12) and *in vivo* results based on mouse models with increased or decreased levels of 24-OHC and 27-OHC (13). In this model, cholesterol synthesis is suppressed by the levels of 24-OHC and to some extent also by the flux of 27-OHC from the circulation into the brain. In Fig. 8*B*, the situation in the brain of the *Pdgfb^ret/ret^* mouse is shown. The flux of the suppressor 24-OHC from the brain into the circulation is increased, leading to reduced inhibition of cholesterol synthesis. This effect is counteracted by the flux of cholesterol from the circulation into the brain. In the untreated *Pdgfb^ret/ret^* mouse, the stimulatory effect of the reduced level of 24-OHC appears to be more important than the inhibitory effect of the flux of cholesterol. After treatment with dietary cholesterol, increasing the flux of cholesterol into the brain and increasing the suppression of cholesterol synthesis, the overall effect of the leaking blood-brain barrier was a normalization of the rate of cholesterol synthesis.

The mechanism behind the normal flux of 24-OHC from the brain to the circulation has not been clarified in detail. The presence of a hydroxyl group in the steroid side chain leads to reordering of phospholipids in biomembranes, allowing a flux of such steroids across the membranes orders of magnitude faster than cholesterol (32, 33). This fact, in combination with a very high concentration gradient between the brain and the circulation, about 100-fold (20), is likely to reduce the need for active transport of 24-OHC across biomembranes. Evidence has been presented from *in vitro* studies that the scavenger receptor class B, type I, is of some importance for the efflux of 24*S*-OHC from cultured endothelial cells (34). According to *in vivo* studies with mouse models, also the transporter Abcg1 and Abcg4 may be of some importance for efflux of side-chain oxidized oxysterols from the brain (35).

It should be noted that the mRNA levels of scavenger receptor B1 (*Srb1*) and *abcg1* were similar in the brains of the mutant mice and the control mice. Both the mRNA levels and protein levels corresponding to *Abca1* were, however, slightly increased in the brains of the mutants. In this regard, it is of interest that one important function of *Abca1* in the brain is likely to be a transport of cholesterol from astrocytes to neuronal cells (36, 37). Given the increased flux of 24OH from the neuronal cells in the present mouse model, there is a need for a higher efflux of cholesterol from the astrocytes to the neuronal cells. From this point of view, the up-regulation of *Abca1* in our mouse model may reflect a compensatory mechanism. Up-regulation of *Abca1* may be a consequence of LXR activation. However, several other LXR-dependent genes were not up-regulated in the brains of the mutant mice, and we are unable to identify the factors behind up-regulation of *Abca1*.

Plant sterols, to some extent, accumulate in the brain, and the possibility has been excluded that the presence of these steroids in the brain is due to a contamination of blood (23). Part of this accumulation is likely to be a consequence of the fact that they cannot be eliminated by the same mechanism as cholesterol by 24(*S*)-hydroxylation (23). Increased permeability of the BBB would be expected to lead to higher levels of the plant sterols in the brains of the *Pdgfb^ret/ret^* mice than in the controls. In accordance with this, the levels of the two plant sterols campesterol and sitosterol were significantly higher than in the controls. The degree of accumulation of campesterol is markedly higher than that of sitosterol. High accumulated levels of these two plant sterols in the mouse brain has been observed also in previous work (23).

There is a continuous flux of the oxysterol 27-OHC from the circulation into the brain, and this flux has been shown to increase in patients with a defective blood-brain barrier (38). In view of this, higher levels of 27-OHC would be expected in the brains of the mutated mice as compared with the controls. This was not found, however. Part of the explanation may be that, similar to 24-OHC, 27-OHC may also leak out from the brain into the circulation in the *Pdgfb^ret/ret^* mouse. Most 27-OHC present in the brain originates from the circulation (6). The brain contains some sterol 27-hydroxlase, however, and thus there may be some production of 27-OHC also in this organ. 27-OHC formed in the brain of a mouse treated with cholesterol-*d*_6_ would not be expected to contain deuterium. The finding that the ratio between unlabeled and labeled 27-OHC in the circulation of the mutant mouse treated with cholesterol-*d*_6_ was slightly higher than the corresponding ratio in 27-OHC in the corresponding control mouse is consistent with some flux of 27-OHC from the brain into the circulation. Another possible explanation for the failure to find increased levels of 27-OHC in the brains of the mutant mice may be increased metabolism. The oxysterol 7α-hydroxylase CYP7B1 is the most important enzyme for the metabolism of 27-OHC. The mRNA levels corresponding to this enzyme were increased in the brains of the mutant mice, a finding consistent with the hypothesis of increased metabolism.

The relevance of the present results for the human situation is difficult to evaluate. Very recently, a mutation in the human PDGF receptor B gene was described, causing calcification of basal ganglia and cognitive defects (39). Also, the present mouse model has been shown to have calcifications in basal ganglia (39). Thus far, no disturbances in cholesterol homeostasis have been described in the patients with a mutation in the human PDGF receptor B gene. In a population of patients with a defective blood brain barrier secondary to different diseases, no significant increase in levels of 24-OHC was observed, however (38). Thus, it is possible that these clinical conditions are not associated with the same severe effects on the blood-brain barrier as in the present mouse model.

To summarize, we have shown that pericyte deficiency leads to increased flux of cholesterol and plant sterols from the circulation into a mouse brain. Unless the cholesterol levels in the circulation are increased, this flux is not sufficient to down-regulate cholesterol synthesis in the brain. The leaking blood-brain barrier leads to loss of the regulating oxysterol 24*S*-OHC from the brain into the circulation, resulting in increased cholesterol synthesis. This stimulatory effect can, however, be counteracted by increasing the concentration of cholesterol in the circulation, which is likely to lead to higher flux into the brain.

## Footnotes

3 A. A. Saeed, G. Genové, T. Li, D. Lütjohann, M. Olin, N. Mast, I. A. Pikuleva, P. Crick, Y. Wang, W. Griffiths, C. Betsholtz, and I. Björkhem, unpublished observations.

## Footnotes

2 The abbreviations used are:

BBB: blood-brain barrier
24-OHC: 24-hydroxycholesterol
24S-OHC: 24(*S*)-hydroxycholesterol
27-OHC: 27-hydroxycholesterol
BisTris: 2-[bis(2-hydroxyethyl)amino]-2-(hydroxymethyl)propane-1,3-diol.
